# Development of Protective Autoimmunity by Immunization with a Neural-Derived Peptide Is Ineffective in Severe Spinal Cord Injury

**DOI:** 10.1371/journal.pone.0032027

**Published:** 2012-02-14

**Authors:** Susana Martiñón, Elisa García, Gabriel Gutierrez-Ospina, Humberto Mestre, Antonio Ibarra

**Affiliations:** 1 Centro de Investigación del Proyecto CAMINA A.C., Mexico City, Mexico; 2 Facultad de Ciencias de la Salud, Universidad Anáhuac México Norte, Huixquilucan, Estado de México, Mexico; 3 Departamento de Biología Celular y Fisiología, Instituto de Investigaciones Biomédicas, Universidad Nacional Autónoma de México, Mexico City, Mexico; University of South Florida, United States of America

## Abstract

Protective autoimmunity (PA) is a physiological response to central nervous system trauma that has demonstrated to promote neuroprotection after spinal cord injury (SCI). To reach its beneficial effect, PA should be boosted by immunizing with neural constituents or neural-derived peptides such as A91. Immunizing with A91 has shown to promote neuroprotection after SCI and its use has proven to be feasible in a clinical setting. The broad applications of neural-derived peptides make it important to determine the main features of this anti-A91 response. For this purpose, adult Sprague-Dawley rats were subjected to a spinal cord contusion (SCC; moderate or severe) or a spinal cord transection (SCT; complete or incomplete). Immediately after injury, animals were immunized with PBS or A91. Motor recovery, T cell-specific response against A91 and the levels of IL-4, IFN-γ and brain-derived neurotrophic factor (BDNF) released by A91-specific T (T_A91_) cells were evaluated. Rats with moderate SCC, presented a better motor recovery after A91 immunization. Animals with moderate SCC or incomplete SCT showed significant T cell proliferation against A91 that was characterized chiefly by the predominant production of IL-4 and the release of BDNF. In contrast, immunization with A91 did not promote a better motor recovery in animals with severe SCC or complete SCT. In fact, T cell proliferation against A91 was diminished in these animals. The present results suggest that the effective development of PA and, consequently, the beneficial effects of immunizing with A91 significantly depend on the severity of SCI. This could mainly be attributed to the lack of T_A91_ cells which predominantly showed to have a Th2 phenotype capable of producing BDNF, further promoting neuroprotection.

## Introduction

Protective autoimmunity (PA) is a physiological T cell-dependent response to neural antigens that protects rather than destroys neural tissue [1]–[5]. In order to obtain the maximal benefits of PA, it is possible to boost this response by immunizing with neural constituents. However, when immunizing with self-antigens there is always a risk of developing a pathological autoimmune response. At the moment, PA can be boosted with non-encephalitogenic peptides such as A91, these have significant evidence of their neuroprotective effects after SCI. A91 is a peptide derived from myelin basic protein (sequence 87–99) but replacing the lysine residue at position 91 with alanine. Immunizing with A91 reduces tissue damage and improves motor recovery after moderate SCI. The therapeutic effect of this strategy has been improved by combining A91 immunization with other therapies [6]. As immunizing with these types of peptides promises important expectations in the future, it is relevant to better understand the general conditions needed to achieve its beneficial effects.

The effect of PA depends on the rapid and proper development of the specific immune response [7] in such a way that any disturbance could derail the benefits of PA. A previous study in our laboratory showed that the adaptive immune response to any immunogen depends on the severity of SCI [8]; the more severe the injury (severe SCC) the more impaired the immune response. Since the beneficial outcome of PA depends on the proper development of this response we attempted to evaluate motor recovery, lymphocyte proliferation, T cell phenotype and neurotrophin production in animals subjected to SCI. Injury consisted of either a moderate SCC, severe SCC, incomplete SCT or complete SCT. A91 induced a better motor recovery in rats with moderate SCC. This beneficial effect was associated with a proliferative T cell response to A91 and the subsequent improvement in motor recovery. The proliferative response was characterized by a Th2 phenotype and the significant release of BDNF. In contrast, severe injuries eliminated the beneficial effect of A91 on motor recovery. This failure was associated to the decrease of the specific immune response towards A91.

## Materials and Methods

### Animals

Adult female Sprague-Dawley rats (13–14 weeks old, 200–220 g) were supplied by the Animal Breeding Center of Proyecto Camina A. C. Efforts were made to minimize the number of animals used and their suffering.

### Ethics Statement

All procedures were in accordance with the National Institutes of Health (US) Guide for the Care and Use of Laboratory Animals and the Mexican Official Norm on Principles of Laboratory Animal Care (NOM 062-ZOO-1999). All animal procedures were approved by the National Council of Science and Technology of Mexico (CONACYT) Animal Bioethics and Welfare Committee (ID: 57204; CSNBTBIBAJ 090812 960).

### Spinal Cord Injury

Rats were anesthetized by intramuscular injection of ketamine (80 mg/kg; PISA Laboratories, Mexico City, Mexico) and xylazine (12.5 mg/kg; Bayer Laboratories, Mexico City, Mexico). One hour after anesthesia induction the spinal cord was exposed and the animals were subjected to a SCC or SCT at T9. For contusion, a 10-g rod was dropped onto the exposed spinal cord from a height of 25 (moderate) or 50 mm (severe), using the NYU impactor (NYU, New York). This device is shown to inflict a well-calibrated contusive injury of the spinal cord [9]. Prior to transection, the dura mater was dissected and separated from the spinal cord with a 30-gauge needle. Complete transection was performed by sliding a straight-edged scalpel blade through the spinal cord. Accuracy of the injury was visually verified by passing a micro-hook through the internal contour of the dura. For incomplete transection, approximately 50% of the dorsal spinal cord (including the corticospinal tracts) was transversely cut with iridectomy scissors. After injury, the aponeurotic plane and the skin were sutured separately with nylon thread.

### Animal Care

The animals were matched for age and weight in each experiment and housed in pairs in a light- and temperature-controlled room. To minimize stress, animals were handled daily at least once a day 7 days prior to the surgical procedure.

Sterile bedding and filtered water was replaced daily. Bladder expression was assisted manually twice a day until automatic voidance was regained. During the first week after contusion the animals received a course of enrofloxacine (Marvel, Mexico City, Mexico) in their drinking water at an approximate dose of 64 mg/kg/d. All rats were carefully monitored for evidence of postsurgical complications. Animals with signs of infections were excluded from the study.

### Antigen

The A91 peptide was derived from the encephalitogenic sequence of myelin basic protein (MBP; amino acids 87–99). Nonencephalitogenicity was obtained by replacing the lysine residue at position 91 with alanine. The modified peptide was purchased from Invitrogen Life Technologies (San Diego, CA, USA). Reverse-phase HPLC confirmed that the purity of the A91 peptide was >95%.

### Active immunization

Rats were immunized subcutaneously at the base of the tail with 150 µg of A91 or phosphate buffered saline (PBS) emulsified in an equal volume of complete Freund's adjuvant (CFA) containing 0.5 mg/ml *Mycobacterium tuberculosis* (Sigma, St. Louis MO) within a 60 min time frame after injury.

### Assessment of functional recovery

Motor functional recovery was assessed using the Basso, Beattie & Bresnahan (BBB) open-field test of locomotor ability [10]. The recovery was scored on the BBB Locomotor Rating Scale of 0 (complete paralysis) to 21 (complete mobility). Animals were tested weekly during an 8-week period by observers blinded to the treatment received by each rat.

### T cell proliferation

Cells were pooled from excised inguinal lymph nodes 14 days after SCI (n = 4). The cells were cultured in quintuplicate flat-bottomed wells in 0.2 ml of RPMI-1640 medium (GIBCO, New York) supplemented with 10% fetal bovine serum (Gibco, New York) on a 96-well microtiter plate. Cells (2.5×10^5^ cells per well) were cultured 72 hrs in antigen-free medium or together with A91 (10 µg/ml), ovalbumin (OVA; 10 µg/ml; Sigma), or concanavalin-A (ConA; 10 µg/ml; Sigma St. Louis MO) at 37°C in 5% CO_2_. After two washes with RPMI-1640, cells were labeled with carboxifluorescein diester amine (CFSE) (Molecular Probes), CFSE-labeled cells divide and its progeny are endowed with half the number of carboxyfluorescein-tagged molecules and thus each cell division can be assessed by measuring the corresponding decrease in cell fluorescence. CFSE was used at a final concentration of 1 µM and, 5 µl/well of this solution were rapidly dispensed into the cell suspension insuring a homogeneous labeling. Cells were then incubated for 24 h at 37°C. Staining was halted by the addition of an equal volume of fetal bovine serum. The proliferative response was determined by flow cytometry. Cells were also stained with phycoerythrin-labeled anti-CD4 monoclonal antibodies (BD Pharmigen, San Diego, CA), unstained cells were used as controls. Cells stained with CFSE and CD4 were analyzed. For analysis, the area of lymphocytes was selected based on the light scattering characteristics (size/granularity) of these cells. Afterwards, the area of CD4+ cells was selected and analyzed for CFSE fluorescence. Mean fluorescence intensity data was obtained from fluorescence histograms to evaluate the fractions of T cells that have completed certain number of divisions. Ten thousand events were collected for each sample on a FACSCalibur flow cytometer (BD Bioscence, Mountain View, CA) and analyzed using CellQuest Pro software (BD Bioscences). The stimulation index (SI) was calculated by dividing the mean percentage of proliferation in experimental wells by the mean percentage of proliferation in the corresponding control wells (cells cultured without antigen).

### Cytokine analysis

An ELISA kit (BD Biosciences, San Diego CA) was used to analyze the concentration of either IL-4 or IFN-γ in supernatants obtained from T cell proliferation assays as described by the manufacturer.

### BDNF analysis

An ELISA kit (Chemicon International) was used to analyze the concentration of BDNF in supernatants obtained from T cell proliferation assays as described by the manufacturer.

### Statistical analysis

Data was analyzed using the GraphPad Prism 3.0 software and presented as mean ± standard deviation (SD). Motor recovery was analyzed by two-factor ANOVA for repeated measures. The proliferative response was evaluated using the Kruskal-Wallis test with *post-hoc* Mann-Whitney U tests. In the analysis of the IL-4/IFN-γ index and BDNF levels a Student's t-test was performed. Differences of *p*≤0.05 were considered statistically significant.

## Results

### A91-induced motor recovery is impaired by severe SCI

**Figure 1:**
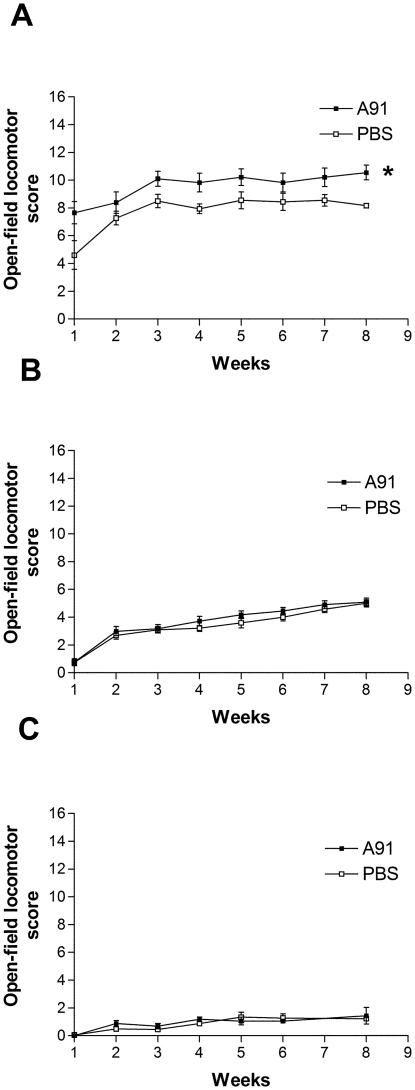
Motor recovery of spinal cord injured rats. Animals were subjected to a moderate (A) or severe spinal cord contusion (B). The recovery of rats subjected to a complete SCT is also shown (C). In all cases, animals were treated either with A91 or PBS. Severe injuries abolished the beneficial effect induced by immunizing with A91. ^*^ Different from PBS group (*p*<0.05, two-way ANOVA for repeated measures). Each point represents the mean ± SD of 10 rats.

The first step of this work, investigated the effect of immunizing with A91 on the functional motor recovery of animals subjected to either a moderate SCC, severe SCC, or a complete SCT (*n* = 10 per group). In all cases, the effect of A91 immunization was compared to PBS immunization controls (*n* = 10, see Table 1). Figure 1A depicts that one week after injury, A91 immunization promoted a better recovery in moderate SCC animals (7.9±0.6 *vs.* 4.2±0.9, A91 and PBS immunization respectively; mean ± SD). Towards the end of study (week 8), A91-treated rats showed a significant improvement in recovery compared to PBS-immunized animals (10.2±0.5 *vs.* 8.3±0.3, A91 and PBS immunization respectively; *p*<0.05, *F = 11.67*). In contrast, A91 immunization did not promote any functional improvement in animals subjected to severe SCC (Figure 1B). At the end of the evaluation period, the recovery of A91-immunized rats (5.09±0.1) did not differ significantly from PBS-immunized controls (5.0±0.2, *p*>0.05, *F* = 0.93). Finally, animals subjected to complete SCT had poor post-injury recovery (Fig. 1C). As shown, both A91- and PBS- immunized rats presented comparable motor recovery that was very low, even towards the end of the study (1.6±0.2 *vs.* 1.1±0.1, A91 and PBS respectively; *p*>0.05,*F* = 0.02).

**Table 1:** Number of animals per group used to evaluate motor recovery.

Groups		Number of animals Immunized with	
	A91		PBS
Moderate SCC	10		10
Severe SCC	10		10
Complete SCT	10		10

SCC: Spinal cord contusion; SCT: Spinal cord transection.

### Severe SCI impairs the ability to develop an adaptive T_A91_ cell response after immunization

**Figure 2:**
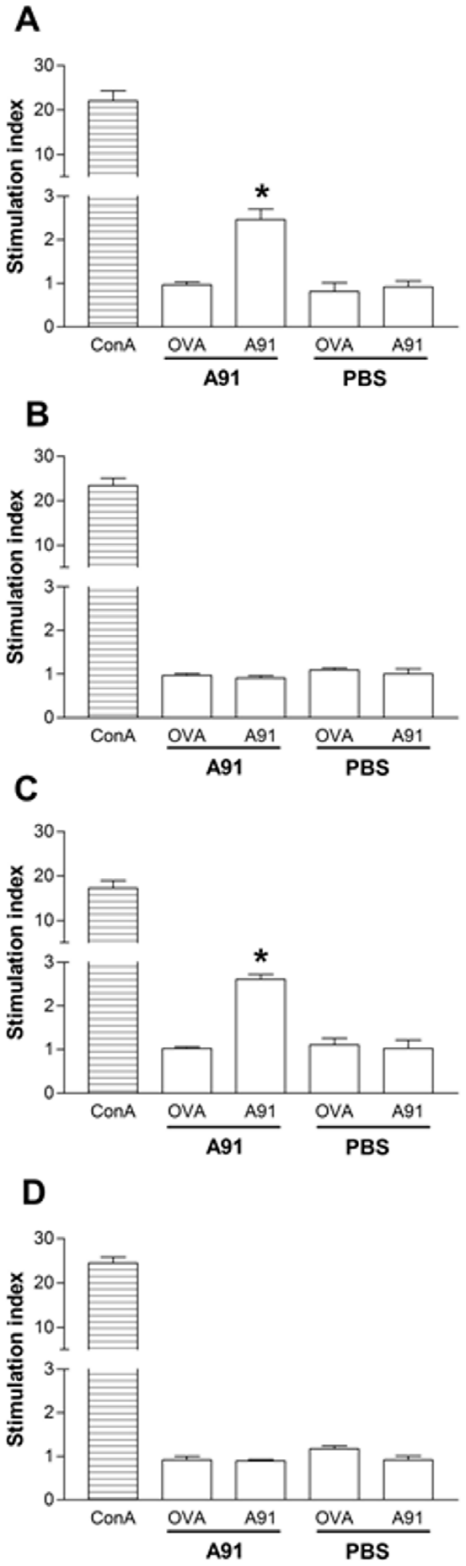
Effect of spinal cord injury on the proliferative response to A91. T cell proliferation against A91 was analyzed in rats with moderate (A) or severe (B) spinal cord contusion (SCC). The same evaluation was performed on rats with incomplete (C) or complete (D) spinal cord transection (SCT). Severe SCC and complete SCT inhibited the proliferative response against A91. ^*^ Different from PBS group (*p* = 0.01, Mann-Whitney U test). Bars represent the mean ± SD of 5 rats.

**Figure 3:**
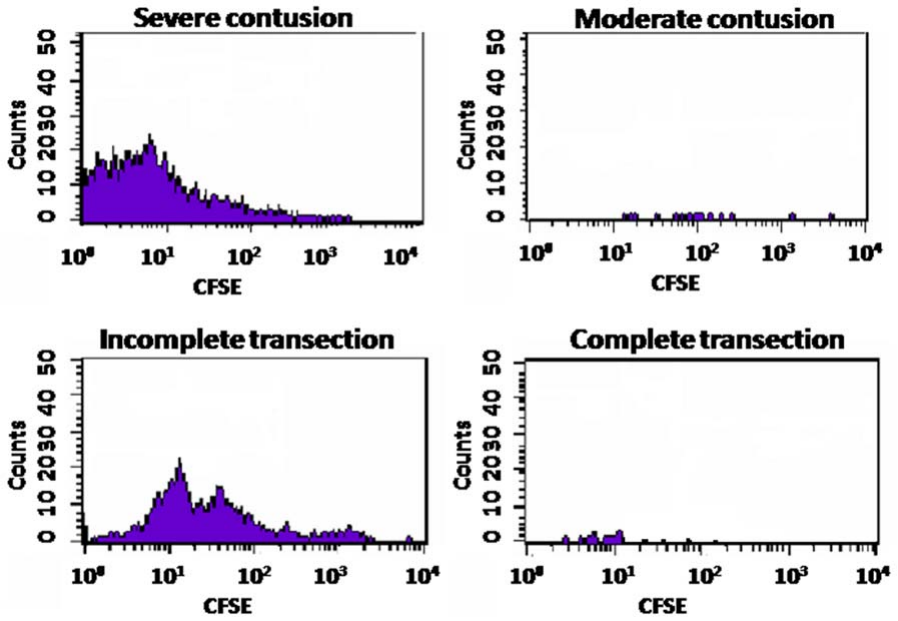
Representative histograms of A91-immunized animals. The ability of T cells to proliferate in the presence of A91 peptide was evaluated using CFSE assays and flow cytometry. Cells stained for CFSE and CD4 were analyzed. Ten thousand events were collected for each sample.

In view of the above results, we now intended to find if immunization with A91 was inducing a specific immune response in animals with severe SCC or complete SCT. The rationale for this study was based on the idea that the absence of neuroprotection could be the result of a failure to induce an anti-A91 response (PA). For this purpose, twenty rats were subjected to a moderate (*n* = 10), or severe (*n* = 10) SCC; another 20 were subjected to a complete (*n* = 10) or incomplete SCT (*n* = 10, see Table 2). The last group was added to investigate the proliferative response in animals that received an incomplete transection of the spinal cord. Immediately after injury, animals were immunized with A91 (*n* = 5) or PBS (*n* = 5, see Table 2). Total leukocyte cell fractions obtained from lymph nodes of these animals were assayed. Figures 2 and 3 show that A91 immunization induced significant proliferation of T_A91_ cells in rats with moderate SCC (Fig. 2A; SI = 2.5±0.42, mean ± SD) or incomplete SCT (Fig. 2C; SI = 2.6±0.2). However, in the case of rats subjected to severe SCC (Fig. 2B; SI = 0.90±0.13) or complete SCT (Fig. 2D; SI = 0.8±0.05), the proliferative response against A91 was absent (see also figure 3).

**Table 2:** Number of animals per group used to evaluate the proliferative response.

Groups		Number of animals Immunized with	
	A91		PBS
Moderate SCC	5		5
Severe SCC	5		5
Complete SCT	5		5
Incomplete SCT	5		5

SCC: Spinal cord contusion; SCT: Spinal cord transection.

### T_A91_ cells have a prevalent Th2 phenotype and secrete increased amounts of BDNF

**Figure 4:**
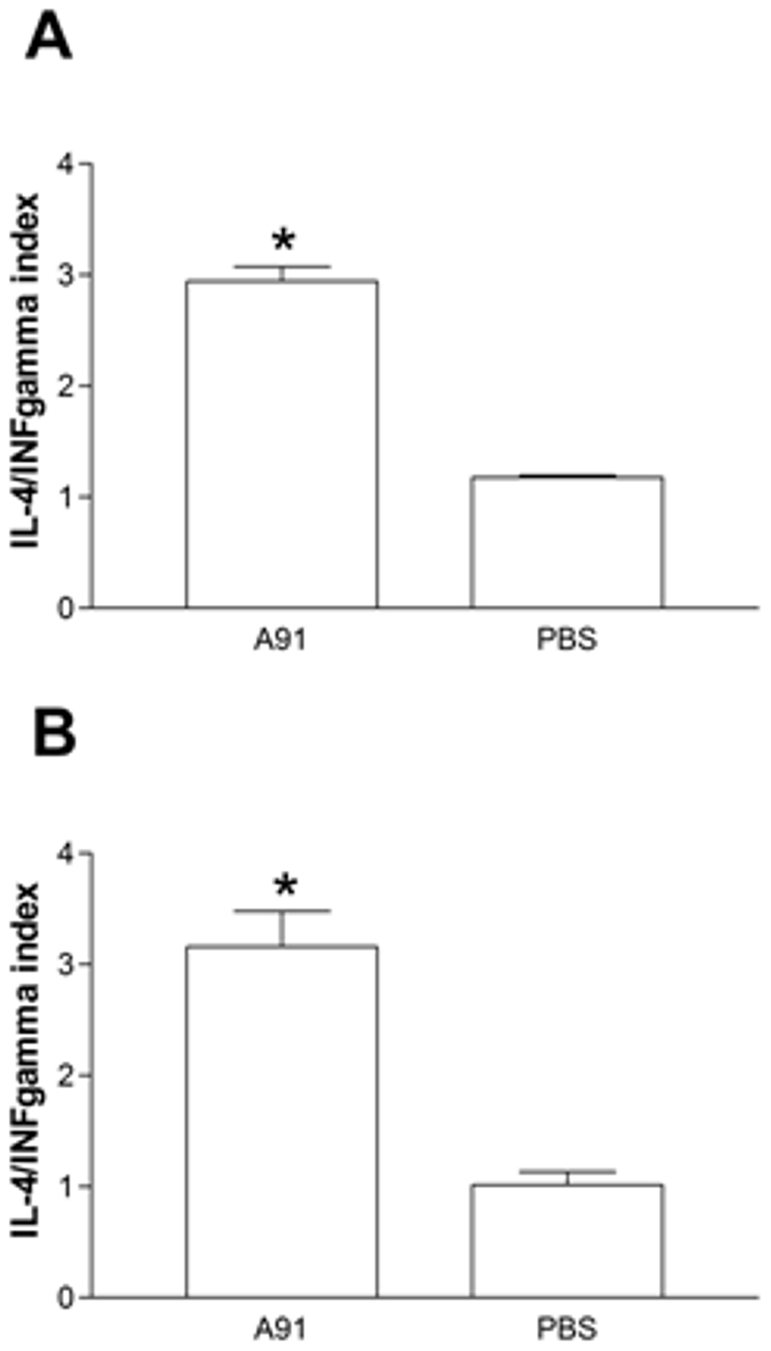
IL-4/IFN-γ index in the supernatant of T cells exposed to A91. Lymphocytes were obtained from rats with moderate spinal cord contusion (A) or incomplete spinal cord transection (B). T_A91_ cells predominantly released IL-4. ^*^ Different from PBS (*p*<0.0001 (A), *p* = 0.001 (B) Student's t-test). Bars represent the mean ± SD of 5 rats. Data was taken from one of three experiments where the same effect was observed.

In order to better characterize the proliferative response induced by A91 immunization in moderately injured animals (rats presenting the neuroprotective effect), we now explored the IL-4/IFN-γ index in the supernatants obtained from the proliferation assays stimulated with A91 peptide and performed to PBS- and A91-immunized animals (previous experiment). Immunizing with A91 caused an increased release of IL-4 in proliferating lymphocytes. In both moderate SCC (Fig. 4A; 2.94±0.3, mean ± SD; *p*<0.0001 *vs.* PBS, Student's t-test) and incomplete SCT rats (Fig.4B; 3.16±0.6; *p* = 0.001 *vs.* PBS, Student's t-test) the IL-4/IFN-γ index of A91-immunized rats was significantly higher than that observed for PBS-immunized controls (1.2±0.2 and 1.01±0.5 respectively).

**Figure 5:**
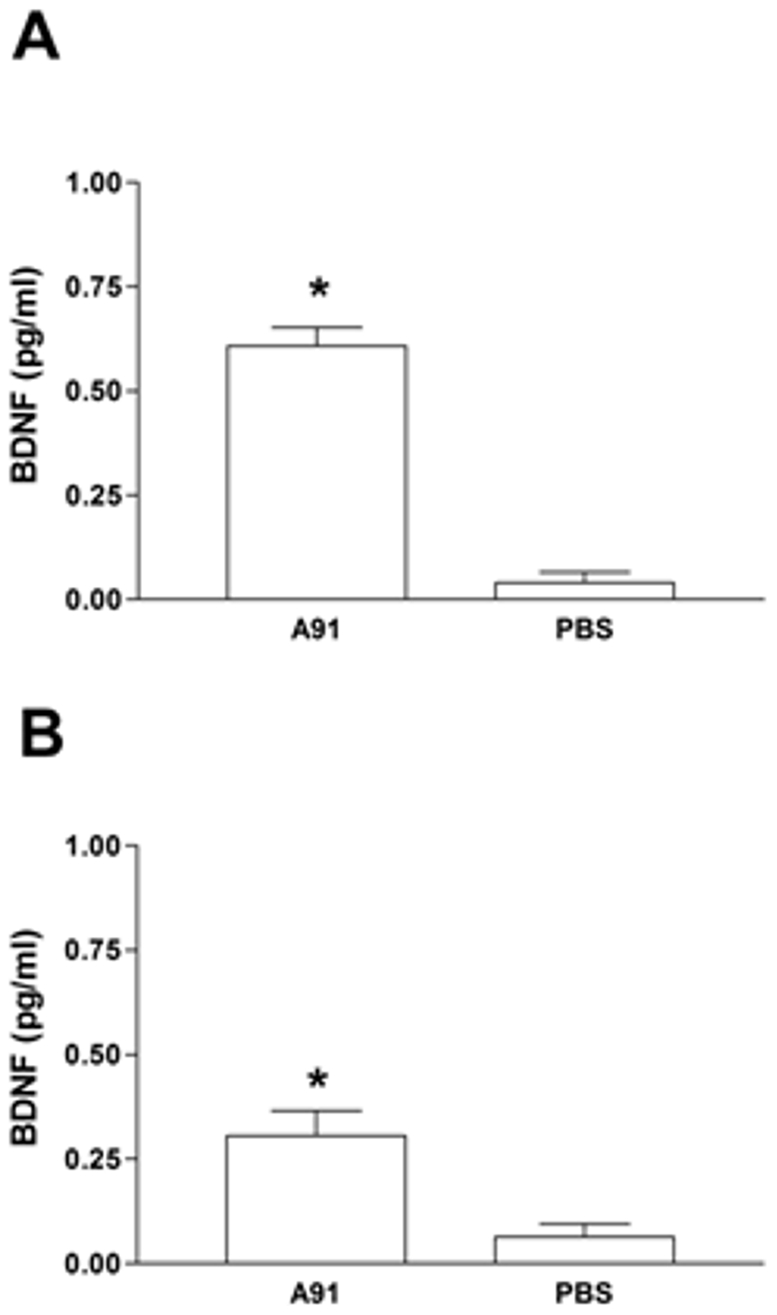
BDNF released by T cells from A91- or PBS-immunized rats. In both cases, T cells were exposed to A91. The levels observed in rats with moderate spinal cord contusion (A) or incomplete spinal cord transection (B) are presented. T_A91_ cells released significant amounts of BDNF. ^*^ Different from PBS (*p* = 0.01 (A), Mann-Whitney U test; *p* = 0.02 (B), Mann-Whitney U test). Bars represent the mean ± SD of 5 rats. Data was taken from one of three experiments where the same effect was observed.

Previous studies have demonstrated that Cop-1- (another neural-derived peptide) specific T cells are capable of producing high levels of BDNF [11]. The next step of this study explored the concentration of BDNF in the supernatant of cells from A91- or PBS-immunized rats that were co-cultured with A91. As is shown, the proliferative response induced by A91 in moderate SCC rats was capable of producing increased amounts of BDNF (Fig. 5A and B). The levels of this neurotrophin were significantly higher in cultures from A91-stimulated animals than those observed in PBS-stimulated controls.

## Discussion

For some time, the immune response has been considered as a harmful mediator after SCI [12]. Nevertheless, there is strong evidence supporting that immune cells improve the neurological outcome after injury [6], [13]. For instance, the immune response developed after immunization with neural-derived peptides (PA) is capable of ameliorating neurotoxicity [14], [15], lipid peroxidation [16] and other degenerative mechanisms [7], [17], [18]. It is clear that immune cells promote a microenvironment where different destructive phenomena are being suppressed, providing tissue protection and improving the functional recovery [2].

Previous studies have shown that immunizing with A91 activates a specific immune response that provides significant neuroprotection and motor recovery after a moderate SCC [6]. In order to better understand this anti-A91 response we investigated the features of PA under different SCI severities. The results indicated that the improvement in motor function observed after moderate SCI was associated with the prevalence of an anti-inflammatory Th2 phenotype. In addition, this Th2 phenotype was capable of releasing significant amounts of BDNF. Previous studies have shown that immunizing with A91 induces a Th2 phenotype in models of experimental autoimmune encephalomyelitis (EAE), in this case the immunization ameliorated the course of EAE [19]. The present work demonstrated, for the first time, that A91 also induces a Th2 phenotype in experimental models of SCI. That finding provides substantial elements to explain, at least in part, our previous observations about the inhibitory effect of A91 immunization on iNOS gene expression and nitric oxide production [20]. Cytokines like IL-4 and IL-10 downregulate the expression of the iNOS gene, we hypothesized that this effect was caused by the action of a predominantly IL-4-secreting Th2 phenotype [21], [22]. On the other hand, the fact that this Th2 phenotype is capable of increasing BDNF production also provides more elements to explain the protective action of A91 immunization. BDNF prevents the death of motor neurons in newborn rats after nerve transection through its action on tyrosine kinase (Trk) receptors [23] and also rescues spinal cord motor neurons from axotomy-induced cell death [24]. The increased production of this neurotrophic factor could also be a relevant mechanism of neuroprotection.

Another significant finding was the reduction of the A91 response after severe SCI. With regard to this, it is worth mentioning that this loss was observed using the same immunization scheme used in moderate SCI. Previous studies in our laboratory have shown that immunizing with 150 µg of A91 is sufficient to obtain neuroprotection [16] and improve functional recovery after a moderate SCI [6]. The present work applied the same scheme and was unable to induce the specific immune response after either a severe SCC or a complete SCT. Moreover, the absence of an A91 response was associated with an impaired motor recovery. The lack of a response could be the result of a state of immunosuppression developed after injury. The existence of a SCI-immune depression syndrome (SCI-ICD) has been reported to develop during the first seven days after injury [25]. Furthermore, our group demonstrated that severe SCC further worsens this state of immunodepression making humoral and cellular responses almost inexistent [8]. The impact of SCI on the immune system is the result of several factors. For instance, immediately after injury there is a period of increased autonomic discharge causing catecholamine over-production. Catecholamines are capable of inhibiting lymphocyte proliferation [26] and cause rapid and extensive apoptosis in lymphoid organs [27]. After injury there is also a significant increase in corticosterone, a steroid hormone involved in regulation of the immune response [28], [29]. According to the above observations, it appears that the scenario induced by SCI does not allow immune cells to be competent enough to undergo adaptive immunity activation. However, several studies (including the present manuscript) demonstrate that in spite of immune system-suppression after injury, the activation of a specific response against an immunogen is still feasible [6], [16], [30], [31]. Nevertheless, the severity of the injury could be a strong determinant in the functioning of the immune system. In light of this notion, and considering the results of our previous work, it is likely that the more severe the SCI, the larger the impairment in immunological function [8]. Under this context, we could explain the failure to induce an anti-A91 response and thus the failure in functional motor recovery in animals with severe injuries.

In view of the present results, it should be stressed that stimulation of PA cannot be performed under the basis of a general paradigm, even if the animals belong to the same strain or share similar weight and age. The previous observation indicates that experimental protocols evaluating PA in animals with severe SCI should consider the altered activation of the adaptive immune response.

Finally, in animals with complete SCT it is difficult to expect that PA (even if it is developed) will exert its protective actions. Since there are no spared axons in complete SCT this model does not benefit from the effects of neuroprotection. Therefore the beneficial mechanisms exerted by PA could be directed towards ameliorating the damage by restoring the neural tissue; however this phenomenon should be further explored.
